# Toward ubiquitous sensing: Researchers turn WiFi signals into human activity patterns

**DOI:** 10.1016/j.patter.2023.100707

**Published:** 2023-03-10

**Authors:** Jianfei Yang, Xinyan Chen

**Affiliations:** 1 Nanyang Technological University, Singapore 639798, Singapore

## Abstract

Jianfei Yang, a principal investigator and postdoc at Nanyang Technological University (NTU), and his student Xinyan Chen have developed a comprehensive benchmark and library for WiFi sensing. Their *Patterns* paper highlights the advantages of deep learning for WiFi sensing and provides constructive suggestions on model selection, learning scheme, and training strategy for developers and data scientists in this field. They talk about their view of data science, their experience with interdisciplinary WiFi sensing research, and the future of WiFi sensing applications.

## Main text

### What would you like to share about your background (personal and/or professional)?

**Jianfei Yang:** Let me introduce myself first. I was born in Binzhou, a quiet city in Northern China. Due to a large number of peer students, it is quite challenging to get into a good university via a college entrance examination and find a path to research. No doubt, I studied very hard from 6 a.m. to 11 p.m. in high school and spent a wonderful time at Sun Yat-Sen University for my undergraduate study. My fate with research and Singapore began in 2016 when I saw a PhD opportunity from my PhD advisor, Prof. Xie Lihua. My advisor is an expert in control and robotics, and this position aims at artificial intelligence (AI) of things (AIoT), which is interdisciplinary. I feel lucky to meet my supervisor and have a very interesting research topic. After my PhD study, I continue to work in interdisciplinary AI research, an AI-aided civil engineering project at BEARS, UC Berkeley. Currently, I am an independent principal investigator (PI) at Nanyang Technological University, conducting my own research on multimodal learning for smart sensing.

**Xinyan Chen:** I am currently a final-year undergraduate student at NTU pursuing a bachelor of engineering in infocommunication engineering. I started the research work 3 years ago guided by Dr. Yang. During these 3 years, I applied deep-learning approaches to realize large-scale WiFi sensing, optimize supervised WiFi channel state information (CSI) classification performance, and estimate human pose with mmWave radar. I also did a 6-month internship at Huawei Singapore research center, where I applied deep-learning techniques to identify non-line-of-sight ultra-wideband (UWB) data. I found my great interest in research on the internet of things (IoT) and wireless sensing and decided to pursue further research in this field.

### What motivated you to become a (data) researcher? Is there anyone/anything that helped guide you on your path?

**JY:** My interest in data science stemmed from my second year of undergraduate study when I met my first advisor, Prof. Chang-Dong Wang at Sun Yat-Sen University. He is a data-mining scientist and has published many high-quality papers on clustering algorithms. Though I was quite young, he recruited me to join his team. He discussed many interesting topics in the data-mining field, which motivated me to explore scientific research. The first topic we discussed was how to build a data model for information diffusion in social networks. He told me that information diffusion is like crowd flow in CCTV cameras that can be captured and can be used to indicate many characteristics of a social network, such as activity level. Looking back on this experience, I found great interest in data research and decided to continue my PhD study after the undergraduate period.

**XC:** I was first introduced to this field when I was a sophomore. I attended an undergraduate research program to apply deep-learning and data science techniques on WiFi CSI data to solve complex tasks in the real world. The key motivation for me to work in this field is that deep-learning and data science techniques empower us with the ability to use radio-frequency signals to tackle complex real-life challenges and provide a non-vision-based approach to solving traditional computer-vision problems.

Dr. Yang plays a significant role in guiding me on my research path. He brought me into this field and taught me various deep-learning and data science techniques. He encouraged me to learn techniques in different fields of deep learning and gave me insights on how to apply these techniques to WiFi CSI data. His optimistic attitude and passion for this field always inspire me to explore new possibilities in our field.

### What is the definition of data science in your opinion? What is a data scientist? Do you self-identify as one?

**JY:** In my opinion, data science is a new subject to study data properties and find specific ways to model the data. It can be regarded as a new powerful tool for other disciplines, such as biology and chemistry. It is seen that many papers in bioinformatics (also published in *Patterns*) adopt cutting-edge data science algorithms and derive interesting findings. Therefore, data scientists should be a group with wide range, coming from multiple disciplines. In this sense, I am also regarded as a data scientist and propose algorithms for WiFi sensing by exploring data patterns in WiFi data.

**XC:** In my view, data science explores the inner logic within the data, which includes but is not limited to the patterns, the characteristics, and the information. A data scientist works on the data to figure out how the inner logic of data could be interpreted and applied to give innovative insights into real-life problems across various fields. To become a data scientist, people need to have an open mind and meticulous observation ability and to think in innovative ways. I regard myself as a beginner in data science and there is a long way to go and much effort to be paid to become a data scientist.

### Aside from supervising research, what do you expect from your supervisors to help you develop into a (data) scientist or researcher?

**XC:** Aside from academic guidance, I would expect my supervisors to help me develop the ability to think and solve problems independently. In the process of scientific research, it is unavoidable to face challenges and predicaments. The keys to overcoming these barriers are to calmly analyze the problem and look at the problem with a positive attitude. I would also expect my supervisors to help me develop teamwork abilities. Complicated work is hard for a person to accomplish alone; it requires all team members put in their efforts and work together. Also, just as Shakespeare said, “there are a thousand Hamlets in a thousand people’s eyes”—different people have different views on the same problem. It will be helpful to listen to others’ suggestions in the research.

### Looking back, what advice would you have given yourself at the start of your career? Is there anything you would have done differently?

**JY:** I am still an early-career investigator, working as a PI for 2 years. I would advise myself to imagine a bigger picture 2 years ago. The reason is that the research vision is the upper bound of a research team. When I started my new role, I made my plan mainly including papers and fundings. However, these are only some result-level plans that should be guided and motivated by a bigger picture.

### Why did you decide to publish in *patterns*? What are your standards for choosing journals?

**JY:***Patterns* is one of the most high-quality journals in the data science field. Normally, I publish my paper in specific-domain journals, such as IEEE and ACM, and I never think about publishing it in a scientific journal that includes a broader range of readers. Nevertheless, when I read some papers in *Patterns*, I find that they attract me a lot, despite different disciplines. Reading these papers benefits me not only at the method level but also in an intuitive way. I chose *Patterns* as it has a broader impact, multidisciplinary scope, and great editors who make the review process smooth. I really enjoy this journey with *Patterns*.

### Did you encounter any particular difficulties, or were there any specific challenges about data, data management, or FAIR data sharing that you dealt with? How did you overcome them?

**XC:** In our work,^1^ we make use of four different open source datasets for comparison. However, different datasets have their own organizing format, saving format, and reading format. Some of them only provide raw data. For better use of these datasets, we reorganized the format of every dataset and performed preprocessing on raw data. We also wrote data reading scripts for every processed dataset to make the them easy to read and easy to implement.

### What is the role of data science in your domain/field? What advancements do you expect in data science in this field over the next 2–3 years?

**JY:** Data science serves as an important tool in WiFi sensing. The development of data science promotes WiFi sensing research significantly. From statistical learning to deep learning, stronger and larger models come into existence and enable better capacity to extract features for higher-level tasks. For example, in my field, statistical features with traditional classifiers (e.g., SVM) only support simple activity recognition, e.g., falling recognition, while deep learning enables more complicated human activities. I think there will emerge more tools for deep learning in multidisciplinary data science over the next 2–3 years. Data scientists will gradually turn their tool from statistical learning to a reasonable integration of both statistical and deep learning.

**XC:** With the development of IoT, the usage of smart devices has grown rapidly. The growth of wireless communication data has become exponential. Other than the basic information communication function, wireless communication data also have some other abilities, such as sensing the environment. Data science plays an important role in digging potential usage of wireless communication data such as WiFi CSI. I expect data science could help to broaden the usage of various kinds of wireless communication data and help us use wireless communication data to tackle more complex sensing problems.

### A lot of data scientists continue their career outside of academia; what is your view on that?

**XC:** Everyone has their reasons for their choices. In my view, data scientists who continue their careers in R&D departments in the industry will focus more on the implementation of a project and how a project could create benefits for the company. While data scientists in academia will pay more attention to the innovation of the idea and the correctness of the theory. The purpose of data science research is ultimately to benefit all mankind. Thus, our society not only needs some data scientists to do ground-breaking work in academia but also needs some data scientist to continuously improve existing works and implement them in the real world as well.

### What’s next for the project? What’s next for you?

**JY:** My next project aims at fine-grained WiFi-based pose estimation for Metaverse applications. Metaverse technology maps human beings from the physical world to a virtual world for the purposes of entertainment, healthcare, and work. To feed this requirement, cameras are usually needed to sense human poses, but due to privacy concerns, cameras are not preferable in households and hospitals. We are studying whether WiFi can be leveraged for human-pose estimation. If so, in the future, the Metaverse world could be simulated by WiFi sensing. With this project, I will focus on my research and then try to transform it into commercial usage for future applications.

**XC:** In the next stage of our project, we will explore applying some more deep-learning techniques to WiFi sensing and try to figure out how could deep-learning models help WiFi sensing play to its strengths under poor environmental conditions (e.g., non-line of sight, poor illuminance, and occlusion). I will continue my research with Dr. Yang in this field and pursue a PhD at NTU in the meantime.

### Your recent published papers in *Patterns* is about a WiFi sensing framework. What are your thoughts on using WiFi sensing products in your daily life?

**JY:** WiFi sensing products will come into existence very soon. IEEE will release the new IEEE 802.11bf standardization that officially includes WiFi sensing into the WiFi standard. In 5–10 years, it is expected that off-the-shelf commodity WiFi infrastructures will adopt the standard, and thus massive WiFi sensing data can be acquired. When it comes to the fields of these products, I believe that security and healthcare are the two most important markets. Firstly, WiFi can help spot the intruder. Compared to the infrared sensor, WiFi signals cannot be seen by intruders even with specific tools, and thus WiFi-based security applications provide a better tool for museums or private households. Secondly, as WiFi-based respiration detection is becoming more and more accurate, home-based health monitoring will be a very good product. As far as I know, hundreds of people die during sleep, and there is no effective way to detect the sudden stop of respiration. Wearable devices such as smart watches also help, but many elderlies do not like to wear these watches. WiFi infrastructures have been ubiquitous, and I think they will be the cost-efficient yet effective choices for home-based respiration detection.

**XC:** With the development of the internet era, more wireless devices need information exchange, which means that more WiFi routers will be deployed in our daily life. Nowadays, scientists have shown that WiFi sensing can be used for occupancy detection, human activity recognition, person identification, human-pose estimation, and many other tasks. In the future, the research work on WiFi sensing will further explore its potential and make it more stable. Moreover, the WLAN sensing standard 802.11bf is expected in 2024. By that time, WiFi sensing technology will begin to develop toward civilian popularization and WiFi sensing products will become a lightweight, low-cost, more broadly applicable, and more private solution for the smart home.

### How do you see the privacy issues of WiFi sensing?

**JY:** This is a very interesting question. In fact, compared to the most common sensing tech in our daily life—camera—WiFi sensing has been much more privacy preserving, as we cannot see a meaningful visible image in WiFi data. However, based on our technology, WiFi-based human-activity monitoring also arouses some privacy concerns. People may worry their WiFi data could be leaked, which means that their daily routine can be obtained by hackers. To this end, we are also studying how to prevent such cases. I have several solutions regarding this concern. Firstly, we can decrease the chances of data leakage by not uploading these data to a cloud server. All the WiFi sensing and analytics are performed in the edge devices. Secondly, we can encrypt the sensing data and perform the data transmission. Thirdly, we should establish rules or instructions to protect user privacy. Different choices of data protocols could be selected by users. Some protocols strictly forbid developers and researchers to use their data, while some may support future research by providing their data.

**XC:** The principle of WiFi sensing is that CSI could describe the environmental influence during the propagation of the wireless signal. And WiFi signals are in the electromagnetic spectrum’s non-visible band. Therefore, WiFi CSI data do not describe the details of private surrounding information and personal information in essence. Compared with vision-based sensing approaches, WiFi sensing approaches could better protect users’ privacy and would be easier to accept because WiFi routers have been widely accepted in home scenarios.

**Figure:**
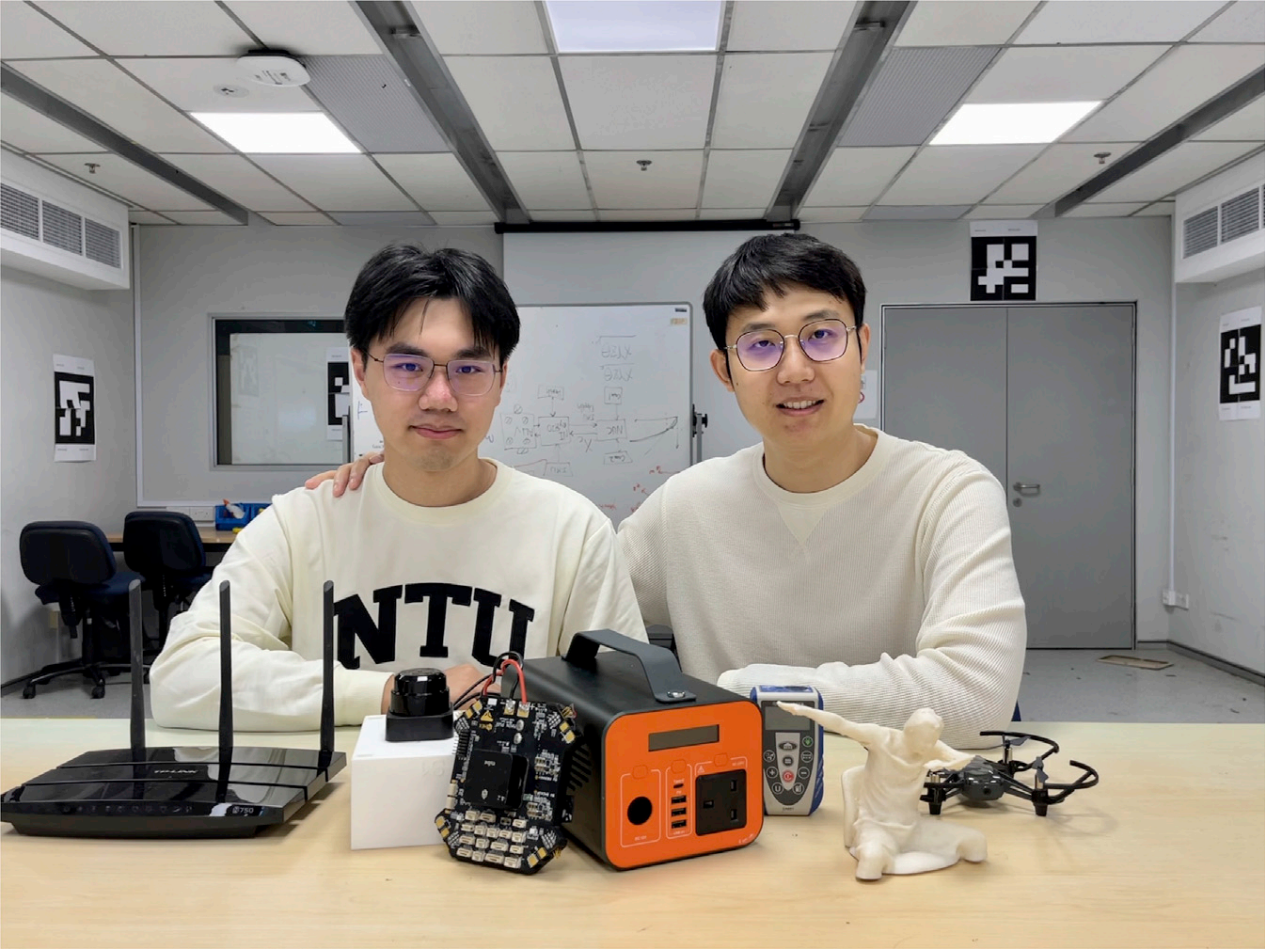
Jianfei (right) and Xinyan (left) at their NTU laboratory, with wireless sensing devices on their table.
